# Secure Scientific Applications Scheduling Technique for Cloud Computing Environment Using Global League Championship Algorithm

**DOI:** 10.1371/journal.pone.0158102

**Published:** 2016-07-06

**Authors:** Shafi’i Muhammad Abdulhamid, Muhammad Shafie Abd Latiff, Gaddafi Abdul-Salaam, Syed Hamid Hussain Madni

**Affiliations:** 1 Faculty of Computing, Universiti Teknologi Malaysia, Johor Bahru, Malaysia; 2 Department of Cyber Security Science, Federal University of Technology, Minna, Nigeria; 3 Kwame Nkuruma University of Science and Technology, Kumasi, Ghana; Bangladesh University of Engineering and Technology, BANGLADESH

## Abstract

Cloud computing system is a huge cluster of interconnected servers residing in a datacenter and dynamically provisioned to clients on-demand via a front-end interface. Scientific applications scheduling in the cloud computing environment is identified as NP-hard problem due to the dynamic nature of heterogeneous resources. Recently, a number of metaheuristics optimization schemes have been applied to address the challenges of applications scheduling in the cloud system, without much emphasis on the issue of secure global scheduling. In this paper, scientific applications scheduling techniques using the Global League Championship Algorithm (GBLCA) optimization technique is first presented for global task scheduling in the cloud environment. The experiment is carried out using CloudSim simulator. The experimental results show that, the proposed GBLCA technique produced remarkable performance improvement rate on the makespan that ranges between 14.44% to 46.41%. It also shows significant reduction in the time taken to securely schedule applications as parametrically measured in terms of the response time. In view of the experimental results, the proposed technique provides better-quality scheduling solution that is suitable for scientific applications task execution in the Cloud Computing environment than the MinMin, MaxMin, Genetic Algorithm (GA) and Ant Colony Optimization (ACO) scheduling techniques.

## Introduction

The Cloud Computing provides computational resources such as the Virtual Machines (VM) to Cloud users on-demand basis [1–3]. Tasks scheduling optimization had been an area of research in IaaS Cloud because it is an NP-hard problem. Nevertheless, the autonomous attribute and the resource heterogeneity within the Clouds and the VM execution necessitate different schemes for task scheduling in the IaaS Cloud computing to be used and tested in order to minimize the makespan time. The makespan time is directly responsible for the tasks execution cost in this environment [4–6].

In recent times, Cloud Computing paradigm is generally employed to convey cloud services over the cyberspace for both scientific and cost-effective resource usage [7]. The capacity of Cloud application and infrastructure services is continuously rising, and hence worse the intricacy of the infrastructures behind the cloud services. Consecutively, to properly manage and supervise such complex infrastructures efficient and secures scientific application scheduling is required [8–10]. When dealing with huge amount of tasks that compete to acquire resources in order to get executed, it becomes clear that an optimal mapping between tasks and resources is considered necessary. This situation is nonetheless *ƝƤ*-complete and consequently sub-optimal results are search as an alternative. Task scheduling in IaaS Cloud is the *ƝƤ*-complete problem. The most notable characteristic of this type of problem is that no fast solution to them is known and also no exact solution is known. *ƝƤ*-complete problems are often addressed by using heuristic or meta-heuristic methods and approximation algorithms [11]. These results are generated by scheduling algorithms (SAs), more often than not as an ingredient of a scheduler. The SA cannot control the distributed systems components directly and are therefore related to broker or agents. Cloud scheduling is meant to solve the problem of task scheduling within diverse environments [12,13].

League Championship Algorithm (LCA) is a population based metaheuristic optimization algorithm that is first proposed by Kashan [14]. To form a synthetic championship setting, the author states some idealized rules to follow, then introduces the promising computational intelligence algorithm that is modeled based on a number of fascinating results relative to sports championship round robin timetable. A detailed survey of the current areas of LCA application is presented by Abdulhamid et al. [15]. The extraordinary increase in the amount of the solution search space produced by the LCA and the superior results produced by the scheme when compared with other metaheuristic algorithms motivates this research to solve scheduling problem in IaaS Cloud computing environment. Therefore, this research presents a novel scientific application tasks scheduling technique for the Cloud computing service using a Global League Championship Algorithm (GBLCA) optimization technique which is a continuation and improvement of our earlier presented research [16,17], but with new improved methods and results. The remaining sections of manuscript is organized as follows: the second Section discuses the related works which includes recent literatures and techniques in scientific application scheduling in Cloud Computing, the third Section explains the global scheduling problem and the fourth Section describes the design process of the proposed GBLCA based application scheduling technique. The LCA’s winner/loser determination feature is detailed in the fifth Section, while the sixth section presents and explains the GBLCA algorithm. The seventh and eighth Sections present the experimental setup and, results and discussion respectively, while the ninth Section chronicles the conclusion and recommendations.

## Related Works

Recently, a number of metaheuristics and basic search techniques have been applied in solving the scheduling problem in IaaS Cloud computing. Metaheuristics can be classified into population-based such as genetic algorithms [18], ant colony algorithm [4] and particle swarm optimization [19]; and trajectory-based such as the simulated annealing [20]. Genetic Algorithm (GA) has been adapted in the recent past to optimize tasks scheduling problems in both grid and Cloud computing environments [21]. GA is a metaheuristic optimization method inspired by the Darwinian evolutionary theory [22,23]. Ga̧sior and Seredyński [24] put forward a multi-objective parallel machine scheduling technique using GA to increase fault tolerance adaptability in the Cloud computing environment. The approach provides not just a single optimal solution, but a set of results that are not subjugated by one another. However, being a multi-objectives scheme, the approach did not show the best method to select the best solution out of the multiple results or solutions produced at the end. Hu and Zhou [25] present a task scheduling technique in IaaS Cloud using the Dynamic Trend Prediction (DTP) and Ant Colony Optimization (ACO). The scheme reserves resource via migration of VM, uses DTP to predict the load adjustment of Cloud datacenter, and then presents the physical balance through regulation. Simulation results indicate that the hybridized technique presented is more capable of improving the performance of datacenter, increase the response speed and precision. Other ACO tasks scheduling schemes in Cloud are presented in [4,26,27], while load balancing awareness can be achieved through scheduling techniques using ACO as presented in [28,29].

Yuan et al. [30] propose a virtual machines scheduling scheme that takes into account the computing power of processing rudiments and consider the computational density of the system. The authors use an improved Particle Swarm Optimization (PSO) to address the VM scheduling problem in the IaaS Cloud computing environment. Verma and Kaushal [31] also present a Bi-Criteria Priority based Particle Swarm Optimization (BPSO) to schedule workflow jobs in a given Cloud computing environment for resources that reduce the execution cost and the execution time under a given deadline and capital. Similarly, the PSO has been adapted in grid and Cloud scheduling to solve the problem of load balancing [32], service selection in grid [33], tunable workflow in Cloud [34] and energy-aware tasks scheduling [35]. The PSO has been utilized widely in Cloud computing systems. Rodriguez and Buyya [36] come out with an approach using PSO to execute scientific workflows on IaaS Clouds, while Pandey et al. [37] apply the PSO in a scheduling heuristic which dynamically balance the job mappings when resources are occupied. However, the PSO is known for its weak local search and slow convergence rate and trapping into local optima when solving complex multimodal problems. A Discrete Symbiotic Organism Search (DSOS) algorithm to minimize the makespan time of jobs scheduling in cloud computing system is also presented by Abdullahi and Ngadi [38]. Symbiotic Organism Search (SOS) is a novel and recently designed metaheuristic algorithm for solving numerical optimization problems. SOS imitates the symbiotic associations demonstrated by organisms in ecology system [39]. Experimental outcome shows that the DSOS performs relatively better than the PSO. The DSOS converges more rapidly in a larger search space which makes it appropriate for extensive scheduling problems. An optimized task scheduling algorithm is introduced using Genetic Simulated Annealing (GSA) technique in Cloud and its implementation [40]. The technique takes into account QoS specifications of various jobs type. The QoS parameters are handled with dimensionless. The scheme proficiently executes the jobs scheduling in the Cloud computing environment [41]. Simulated Annealing (SA) is utilized to calculate the application of a multi-Cloud computing system allocation a workload of tasks with little parallelism but with high arrival speeds and exceedingly variant run-times. The SA technique outperforms the Shortest Queue First (SQF) under both parameters and all their variations. The experiment indicates considerable gains both in performance and cost reduction can be attained via the SA technique in this context [42]. However, the SA is known for its reliance of the solution quality on highest iteration number of the inner loop (cooling schedule) and starting temperature.

MINMIN and MAXMIN [43] are heuristic methods apply to address the problem of task scheduling in Cloud computing. MINMIN heuristic assigns the minimum task earliest from all the accessible tasks and assigns it to a VM that can present the minimum completion time for that job. It enhances the overall completion time of the entire jobs and therefore enhances the makespan time. But it does not reflect on load of the VMs before scheduling as basically transmitting smaller jobs on quicker VMs. At this point, the projected completion time and execution time for a job are measured to be approximately equivalent or close values. The time-consuming jobs have to linger for completing the execution of minor ones. But the technique advances the system’s total throughput [44]. A fault tolerant-aware hybrid heuristic is first proposed to schedule scientific workflows efficiently by Bala and Chana [45]. The hybrid heuristic approach chooses between the MINMIN and MAXMIN under certain conditions, and the concept of maximum child is taken into account. However, complete hybridization of the chosen heuristics is not yet achieved.

## Global Scheduling

Task scheduling in Cloud computing environment takes place at different level of the cloud architecture. At the highest level, there is a task for resource mapping to obtain an optimize schedule and this is called Global scheduling. While at the lowest level, these are also scheduling within the processors which are normally handled by the operating system and it is called as local scheduling. In more technical sense, Global scheduling starts with a complete schedule. The initial schedule can be obtained by different methods, such as randomly selecting a resource for each task, and is further optimized based on the scheduling criteria [46–47]. The techniques usually involve meta-heuristic algorithms such as GA, ACO, and PSO, while in this study the GBLCA is first introduced.

Scheduling tasks in IaaS Cloud Computing system is considered as NP-hard problem of O(m^n^) complexity, where n is the amount of task and m is the amount of resources. As a result, executing time complexity will be exponential to the input size parameters. Hence, researches are now directed towards finding polynomial time approximation metaheuristics for solving this problem, which is fast technique that build schedules whose fitness value is near to the optimum values. In order to achieve the level of optimization needed, the GBLCA based task scheduling technique is developed by modifying the original LCA metaheuristic algorithm inspired based on the metaphor of sports championship in a round-robin sport leagues.

### GBLCA-Based Scientific Application Task Scheduling Technique Design

**Fig 1:**
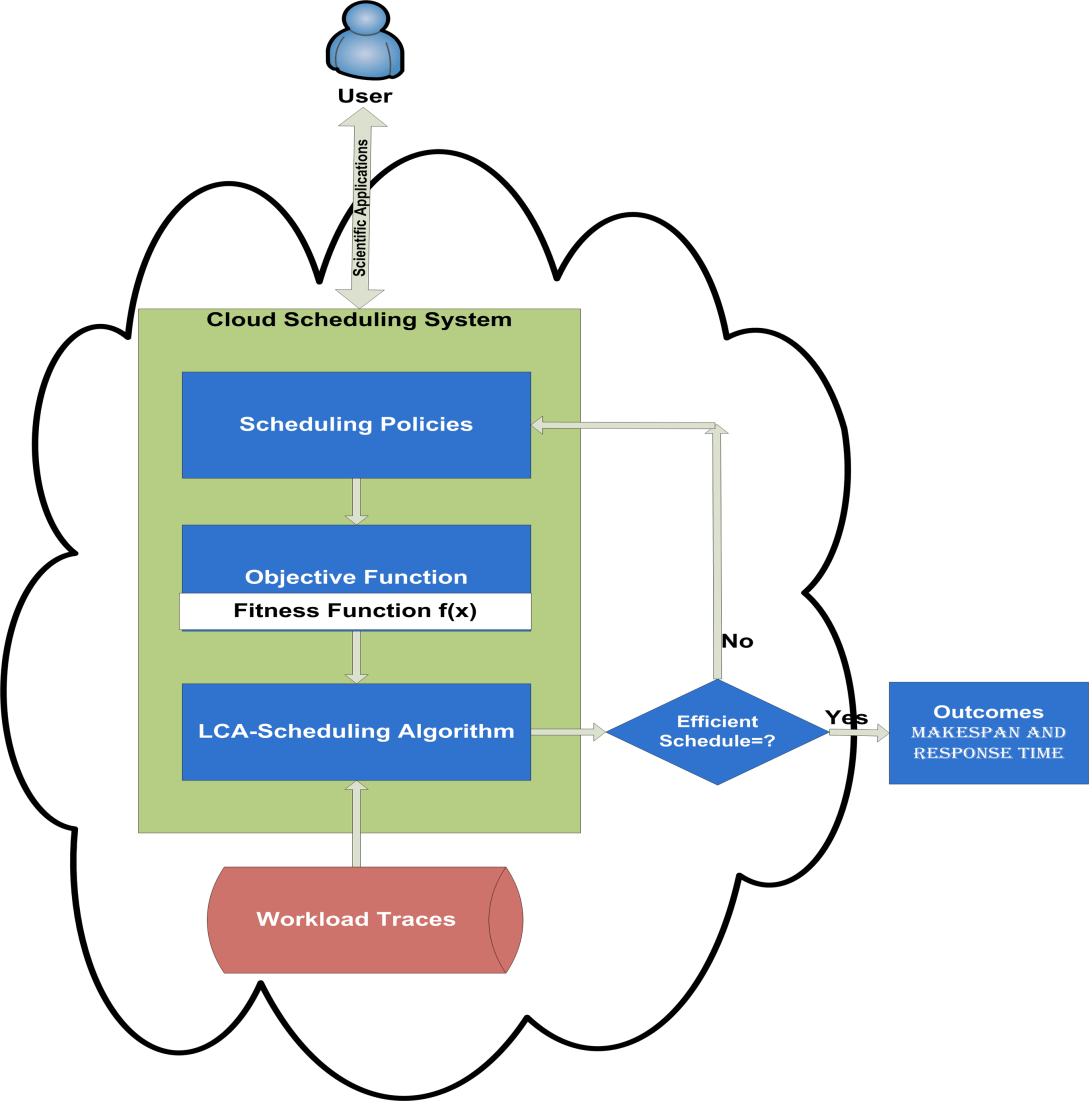
Cloud Task Scheduling Mechanism.

Cloud task scheduling system consists of three key modules; the policies module, the objective function (fitness function) and the scheduling algorithm. Fig 1 shows the interaction of these three important scheduling modules in the proposed design and implementation of the GBLCA-based task scheduling technique in IaaS Cloud environment.

As shown in Fig 1, the first scheduling module of the proposed system is the policy module on which it is based. The scheduling policies are normally set by the Cloud service providers. The scheduling policies are set of rules to establish the Cloud resource allocation and the service level agreements (SLA) for all submitted Cloud applications. Even though, the Cloud computing environment is designed in such a way to give users the illusion of unlimited resources, but in reality is that, there are usually not sufficient resources accessible to satisfy all tasks instantaneously: in effect resource conflict occurs frequently. The scheduling policy is used to settle likely conflicts during tasks scheduling.

The second module of the Cloud scheduling system is the objective or fitness function. It is generally utilized to determine, rank and evaluate the quality of a schedule. As fitness function is given for all task submissions, there is a joint objective function for every schedule. The fitness functions of all allocations together define the optimization problem for the scheduling algorithm. The purpose of the fitness function is to train and test the n tasks and schedule them to the m Cloud resources in order to achieve the minimum makespan possible in an NP-hard problem. The scheduling algorithm is the final module of the proposed Cloud task scheduling system. It has the duty to produce a valid and efficient schedule for the actual stream of submission applications. An efficient scheduling technique is projected to generate optimal schedules with respect to the fitness function within a minimum execution time and consuming only minimum Cloud resources to establish a schedule. In general, the scheduling algorithm must be implementable in a real system. In this research work, a tasks scheduling algorithm using the GBLCA optimization scheme is proposed in order to minimize the makespan in achieving the optimal schedule. The GBLCA scheduling algorithm is fed with workload traces generated from the Parallel Workload Archive or any suitable cloud benchmark dataset [48] to demonstrate its effectiveness.

A fitness function is a particular kind of objective function that is utilized to summarize, as a particular figure of merit, how fairly accurate a specified design solution is to achieving the set objectives. For instance, when using GA optimization technique, each design solution generally corresponds to as a string of records (known as the chromosome). After each iteration of a simulation, the idea is to remove the 'n' worst create solutions, and to form 'n' new solutions from the best design solutions. For every design solution, there is need to rank a stature of value that specify how fairly accurate it came to meeting the general requirement, and this is generated by applying the fitness function to the test, or simulation, outcomes generated from that solution.

Task scheduling problem in the Cloud computing environment can now be formally stated as follows: Given tasks J and resources R, to compute a schedule that assigns each of the tasks J to a specific resource R, in such a way that the cumulative utilization of the tasks on any resource is no greater than the utilization bound of that resource which is 1.0. The task set *J* the resource set *R* and the utilization matrix are measured. Each assignment is modeled as a point in the *m*-dimensional space, where, each coordinate indicate the proportional utilization of the corresponding resource by that task. The features of a task set *J* of size *m* is obtained by clustering them using the Euclidean distance in the *m*-dimensional space, as the distance metric.

The winner/loser determination playing strength or the fitness function is utilized to find the quality of given team solution in the population. The goal of task scheduling technique in the IaaS Cloud computing system is to schedule the n tasks to the m resources (VMs) so as to formulate the tasks within a minimum makespan time. Therefore, the playing strength and fitness of GBLCA algorithm correspond to the makespan time of the schedule.

### Winner/Loser Determination

One of the most important features of the LCA is the winner/loser determination scheme. In this research work, this feature is adequately utilized in determining which job is scheduled on which VM in the IaaS cloud. Considering a normal league system, teams play each other weekly and their game result is evaluated on the basis of win/loss/tie for each of the teams. For instance, in football league, each club is to get three points for a victory, zero for defeat and one for draw. By ignoring irregular abnormalities which may ensure even outstanding clubs in a variety of unsuccessful outcomes, it is probable that a more dominant club having a superior playing pattern defeats the lesser team. In an ideal league situation that is free from uncertainty effects, an assumption can be easily made for a linear correlation between the playing pattern of a club and the result of its matches. Utilizing the playing power condition, the winner/loser decision in LCA is determined in a stochastic approach using criteria that the probability of winning for a club is relative to its degree of fit. Given teams (jobs in our case)*i* and *j* playing a league match at week (time)*t*, with the formations $x_{i}^{t}$ and $x_{j}^{t}$ and playing powers (strength) $f\left(x_{i}^{t}\right)$ and $f\left(x_{j}^{t}\right)$, correspondingly. Let $P_{i}^{t}$ represents the probability of team *i* to defeat team *j* at time *t* ($P_{i}^{t}$ is defined respectively). Given $\hat{f}$ be an ideal value (for example, a lower limit on the best function).

$$\frac{f\left(x_{i}^{t}\right)-\hat{f}}{f\left(x_{j}^{t}\right)-\hat{f}}=\frac{p_{i}^{t}}{p_{j}^{t}}$$ (1)

From the LCA idealized rules, is deduced:

$$p_{i}^{t}+\;p_{j}^{t}=1$$ (2)

From Eqs (1) and (2) above the value of $P_{i}^{t}$ is formulated

$$p_{i}^{t}=\;\frac{f\left(x_{j}^{t}\right)-\hat{f}}{f\left(x_{j}^{t}\right)+\;f\left(x_{i}^{t}\right)-2\hat{f}}$$ (3)

In order to find the winner or loser, a random number in between 0 to1 is generated; if the generated number is $\leq P_{i}^{t}$, it means team *i* won and team *j* lost; else *j* won and *i* lost. This method of finding the winner or loser is in line with the idealized rules. If by chance $f\left(x_{i}^{t}\right)$ approaches $f\left(x_{j}^{t}\right)$, then $P_{i}^{t}$ can be arbitrarily approaching ½. But, if $f\left(x_{j}^{t}\right)$ becomes far $>f\left(x_{i}^{t}\right)$, also written as $f\left(x_{j}^{t}\right)$ » $f\left(x_{i}^{t}\right)$, then $P_{i}^{t}$ tends to one. Then, the value of $\hat{f}$ may be unavailable in the feature, therefore from the best function value found so far (that is, ${\hat{f}}^{t}={\text{min}}_{i=1,\ldots .,L}\left\{f\left(B_{i}^{t}\right)\right\}$.

**Fig 2:**
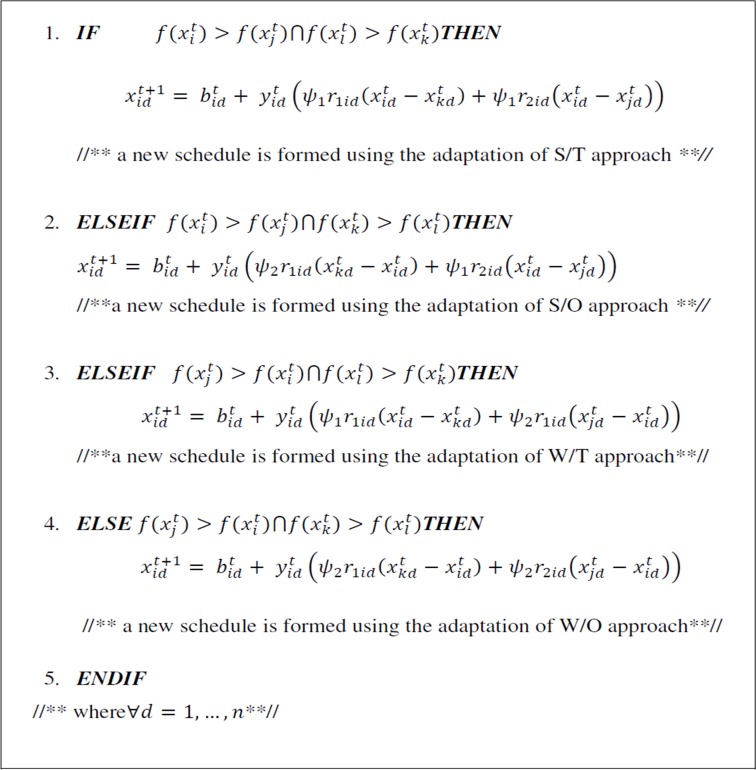
SWOT Pseudo-code.

Using the strengths and weaknesses of each squad player, create a good players combination by taking different constraint into consideration. Likewise, a process is also carried out using artificial analysis method, which is SWOT (i.e strengths, weaknesses, opportunities, and threats) to generate an appropriate focus strategy. Considering that as a rule, clubs play with their recent best formation, while planning the necessary changes suggested from the artificial match analysis; the fresh club formation $x_{i}^{t+1}=\left(x_{i1}^{t+1},x_{i2}^{t+1},\ldots \ldots ,\;x_{in}^{t+1}\;\right)$ for a club *i* where ranges from *i* = 1, ….., *L* at a time t + 1 could be evaluated based on the Fig 2.

The Fig 2 shows that, *d* is the dimension index. *r*_1*id*_ and *r*_2*id*_ are uniform random values between zero and one. *ψ*_1_ and *ψ*_2_ are coefficients that are used to measure the inputs of “retreat” or “approach” mechanisms, in that order. It is also important to note the distinct sign in parenthesis outcomes increase in the direction of the winner or retreat away from the loser.

After the analysis has been done, to generate a fresh formation, the random number of changes made in $B_{i}^{t}$ can be calculated using Eq (4).

$$B_{i}^{t}=\left[\frac{\text{ln}\left(1-\left(1-{\left(1-p_{c}\right)}^{n-q_{0}+1}\right)r\right)}{\text{ln}\left(1-{\text{p}}_{\text{c}}\right)}\right]+\;q_{0}-\;1,\;q_{i}^{t}\in \left\{q_{0},\;q_{0}+\;1,\ldots .,n\right\}-$$ (4)

where *r* represent the random number generated between zero to one and *p*_*c*_ < 1, *p*_*c*_ ≠ 0 donating a controlling variable.

## Global League Championship Algorithm

Population based metaheuristics local search optimization techniques are a increasingly gaining attention from researchers in this new paradigm area of optimization. There are many nature-inspired optimization schemes fitting into this family that mimic metaphors as a funnel in solving NP-hard problems. Some of the most popular members of this family are GA that utilizes the allegory of genetic and evolutionary theory of fitness selection for reproduction to search for solution spaces. Others include the ACO, PSO and SA. Motivated by natural, social and sporting phenomena, metaheuristic optimization techniques have attracted many scientists from a range area of science and technology in recent times. Alongside these applications such as commerce, manufacturing, and engineering, a new metaheuristic algorithm is introduced that applies a novel metaphor as a funnel for solving NP-hard optimization problems.

**Fig 3:**
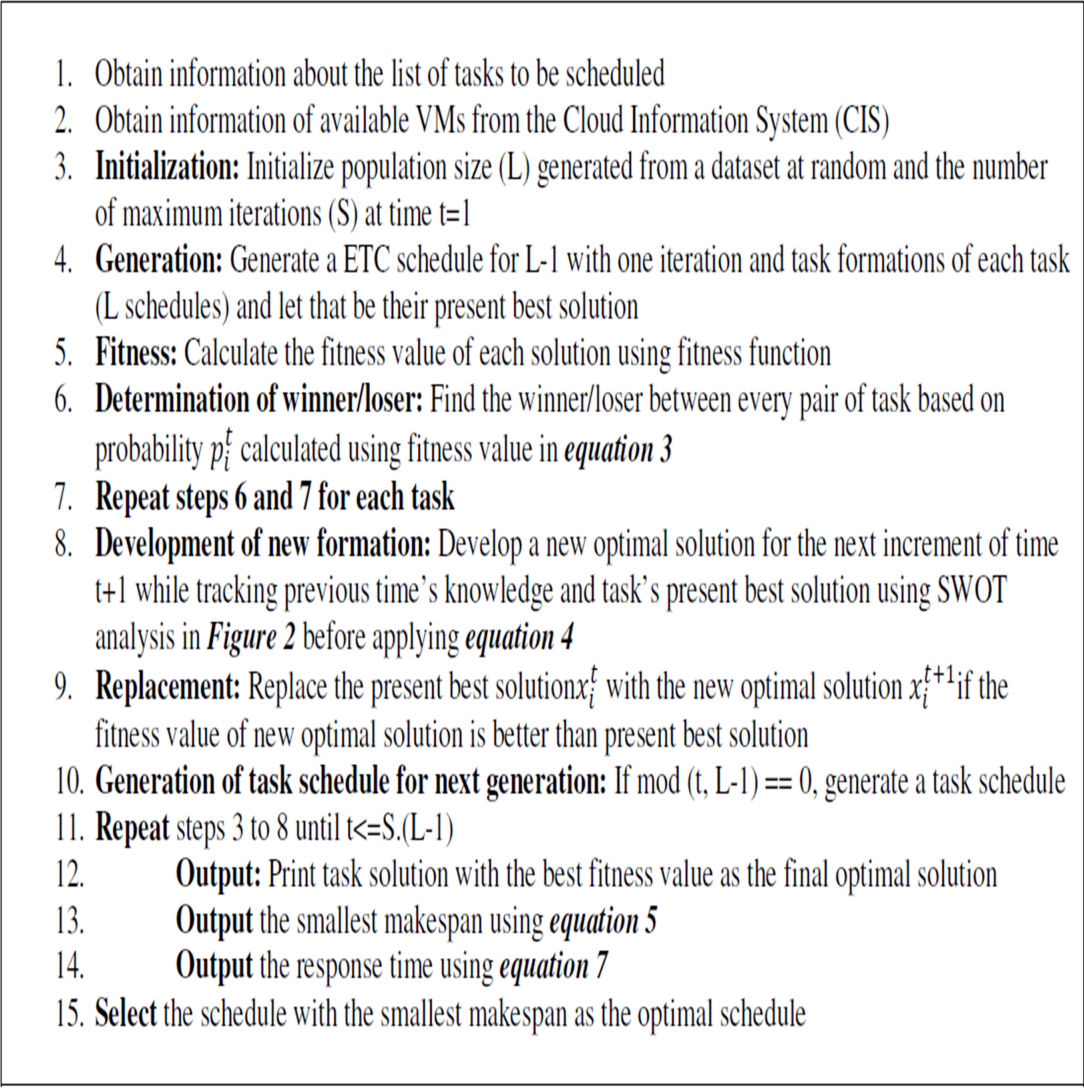
Global League Championship Algorithm (GBLCA).

**Fig 4:**
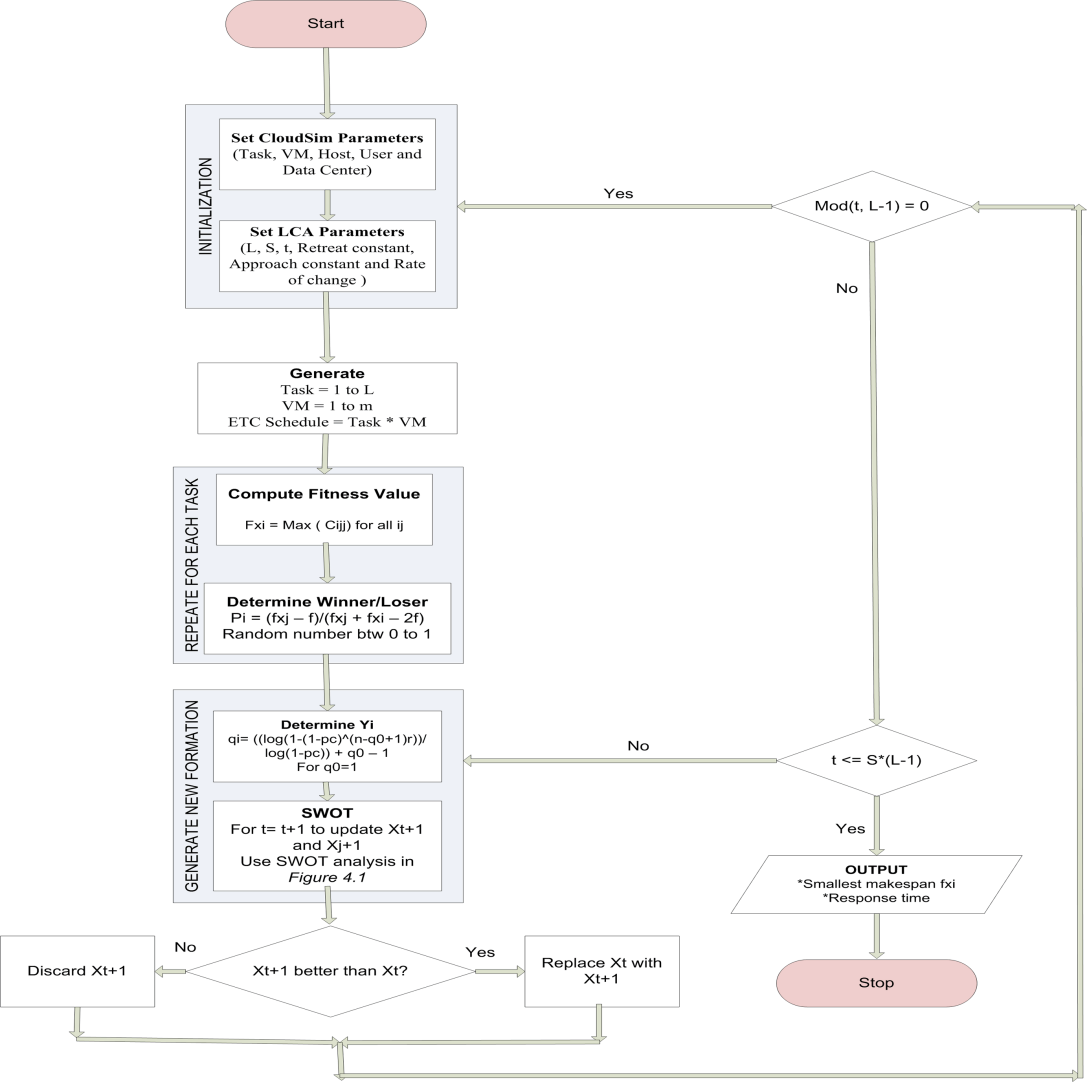
Flowchart of GBLCA Algorithm.

The LCA imitates the sport league championships schedule. Numerous individuals making role as teams participate in an artificial league for a number of weeks (iterations). Using the league schedule in each week, teams compete in pairs and the result is determined in terms of win (1) or loss (0), given known the team’s playing strength (fitness value) resultant from a particular team formation (solution). Keeping record of the preceding week experiences, each team formulates the essential changes in the formation/playing style (a new solution) for the next week contest and the championship goes on for a number of seasons (stopping condition). An example of an incidence matrix related to this problem with six scientific application tasks *T* = {*T*_1_,*T*_2_,…,*T*_6_} and six VMs represented as *V* = {*V*_1_,*V*_2_,…,*V*_6_} resources.The detail of the pseudo-code of the GBLCA is listed in Fig 3, while the flow chart is presented in Fig 4.

## Materials and Method

This section describes the methodology used for the performance evaluation of proposed algorithm and discusses experimental results.

### Experimental Setup

The proposed GBLCA is designed and developed to address the issues of global tasks scheduling in the cloud computing environment. To analyze and evaluate the performance of the algorithm (through simulations) in order to measure the efficiency of GBLCA scheme, the makespan and response time parameters are considered. Therefore, the main purpose of this section is to evaluate and test the performance of GBLCA. The experiments are conducted repeatedly up to 50 times and the average is computed and used with comparable results. Table 1 presents the parameter settings of the selected scheduling techniques. The parameter values of the ACO are based on [29, 50], while the parameter values for the GA are based on [24, 51]. The parameter settings for the GBLCA are based on [14].

**Table 1:** Parameter Values of Scheduling Algorithms.

S/No.	Scheduling Algorithm	Parameter	Value
1.	ACO	Number of ants in colony	10
		Evaporation factor ρ	0.4
		Pheromone tracking weight α	0.3
		Heuristic information weight β	1
		Pheromone updating constant Q	100
2.	GA	Population size	1000
		Maximal iteration	1000
		Crossover rate	0.5
		Mutation rate	0.1
3.	GBLCA	Retreat constant *ψ*_1_	0.5
		Approach constant *ψ*_2_	0.5
		Rate of change p_c_	0.01
		League size *L*	1000

The experimental simulations are implemented with 10 datacenters containing 50 VMs and 200–2000 tasks under the CloudSim simulation environment. The length of the cloudlet is from 800000 MI (Million Instructions). The parameter settings of cloud simulator are presented in Table 2. A simulation assumption is made that tasks are mutually independent, no priority constraint between tasks during execution and non-preemptive.

**Table 2:** Experimental Parameters Setting of Cloudsim.

S/No.	Entity Type	Parameter	Value
1.	User	Number of user	50
		Broker	10
2.	Task	Number of tasks	200–2000
		Length	800000
		File Size	600
3.	Host	Host Memory (RAM)	2048BM
		Host Storage	1000000
		Host Bandwidth	10000
4.	Virtual Machine (VM)	Number of VMs	50
		Type of Policy	Time_Shared
		VM RAM	512BM
		Image Size	10000BM
		VMM	Xen
		OS	Linus
		Number of CPUs	1 on each
5.	Datacenter	Number of Datacenter	10
		Number of Hosts	10

### Parallel Workload Archive

Job traces are generated from the Parallel Workload Archive [48] which contains 73,496 jobs. This workload archive is made available by San Diego Supercomputer Center (SDSC) and is in the Standard Workload Format (SWF) recognized by the CloudSim simulator. This general log encloses information on the user, account, and application, requested and used nodes and time, CPU time, submits, wait and run times. The workload log from the SDSC SP2 is graciously provided by Victor Hazlewood [49], who also helps with background information and explanation.

### Performance Metrics

The performance evaluation metrics of the makespan and the response time are considered. The following definitions are given for usage in the IaaS Cloud environment.

#### Makespan time

The makespan is the maximum completion time or the time when IaaS Cloud system complete the latest job [52–53]. So, if C_ij_ defines the time that resource r_j_ needs to complete job J_i_. Therefore, ΣC_i_ is the total time that resource r_j_ completes all the jobs submitted to it. The Eq (5) defines the makespan in IaaS Cloud environment mathematically.

$$fmax\left(C\right)=\;\text{max}\left\{c_{ij}\;\mathrm{for i jobs mapped to j VM}\right\}$$ (5)

where, *Ci*^’^ is the completion of task *i*. The lesser the makespan the better the efficiency of the algorithm, meaning less time is taken to execute the algorithm.

#### Performance Improvement Rate (PIR) percentage

The PIR is defined as the percentage of performance improvement (or reduction in makespan) for the proposed technique *i* with regards to the other technique *k* and is calculated using the Eq (6).

$$PIR\left(\%\right)=\left(fmax\left(C_{k}\right)-fmax\left(C_{i}\right)\right)\times \frac{100}{fmax\left(C_{i}\right)}$$ (6)

#### Response time

The response time of the Cloud tasks scheduling is influenced by two main factors: (a) The Cloud overhead time which contains the time for preparation, the time of stage in the task, the time to stage out the task, and the time to clean the resources of the Cloud used in the scheduling process. (b) The time of scheduling process which contains the time to execute all tasks in all VMs of the Cloud computing. The response time (RT) can be computed mathematically as

RT=Tprep+Tinmax+Toutmax(n)+Tcleanmax+tmax(pi) (7)

Where;

$$\begin{array}{l} Tprep=\;\text{time needed to prepare the tasks before submitting it to Cloud}\\ Tinmax=\;\text{maximum stage in}\\ Toutmax\left(n\right)=\;\text{maximum stage out}\\ Tcleanmax\;=\;\text{maximum cleaning time}\\ tmax\left(pi\right)=\;\text{maximum search process} \end{array}$$

## Results and Discussion

**Fig 5:**
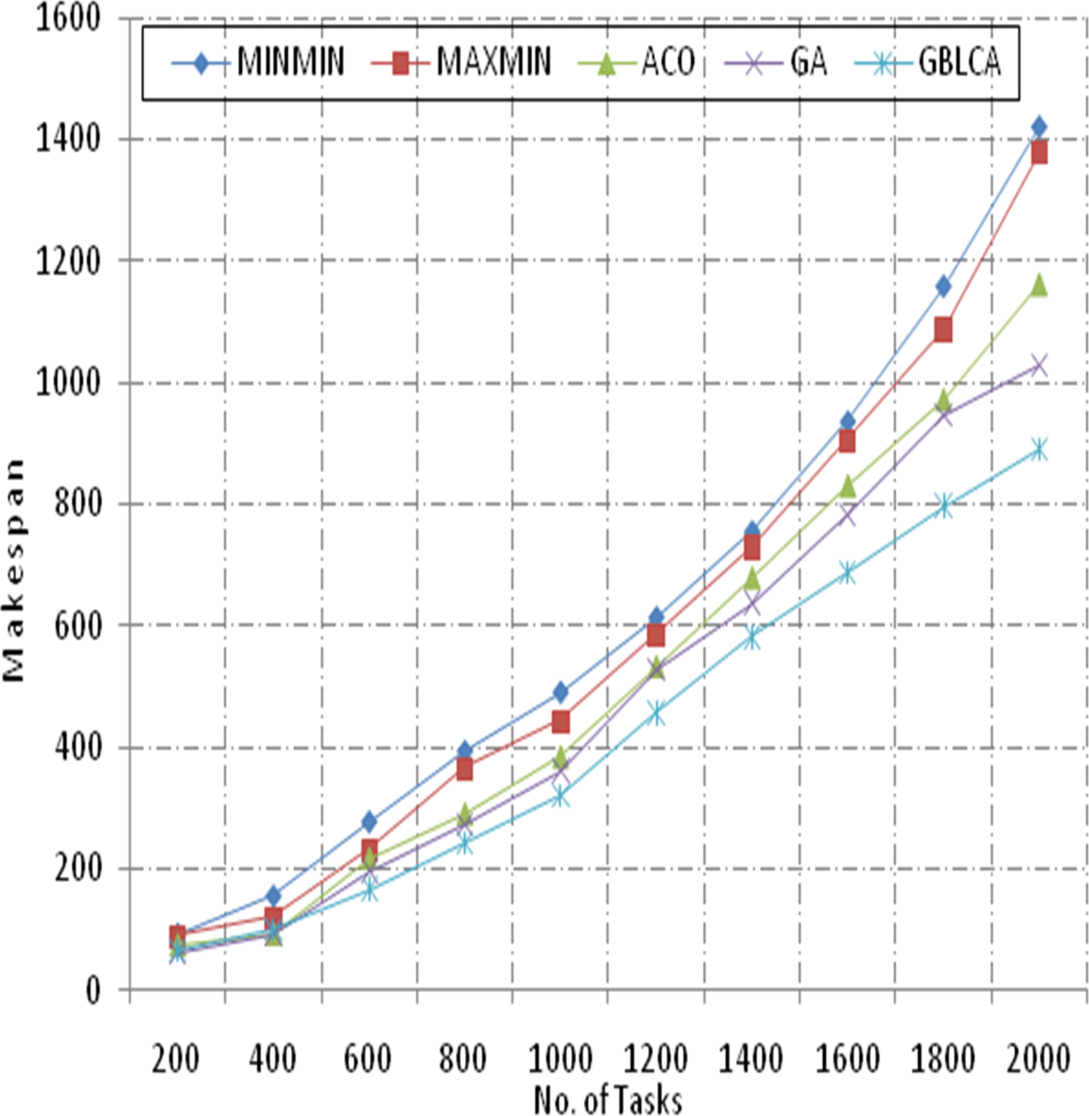
Makespan Time.

Fig 5 presents the makespan time as computed by the five cloud computing scientific applications tasks scheduling algorithms (MINMIN, MAXMIN, GA, ACO and GBLCA). The figure shows that makespan of the scientific applications tasks scheduling techniques increase with the increase number of tasks. The makespan time as computed by the GBLCA scheduling algorithm is lesser than the other four algorithms, especially as the number of tasks increases. The MINMIN has the highest makespan amongst the algorithms under consideration. The results obtained from the CloudSim simulation environment also shows that, GBLCA scheduling algorithm performs moderately better than the MINMIN, MAXMIN, GA and the ACO algorithms throughout the experiment. The implication of this result is that, the proposed GBLCA scheduling technique would help the cloud customers to save more money while using the cloud. This is because the algorithm helps to reduce the makespan time which is the maximum completion time of tasks, making the customers to spend lesser time in the pay per use Cloud Computing environment.

A statistical analysis of the data obtained after 50 trials is presented in the Table 3 in order to assess the significance of the data and robustness of the proposed scheme. Thus, the GBLCA has been run with different number of tasks ranging from 200 to 2000. The best, worst, mean, median and the standard deviation are all computed and presented in the table. The result shows that the minimum, the average, the median, the mode and the maximum values for the 50 runs are very close in each case which also shows significance from the value of the standard deviation. This significance analysis shows that the result follows a normal distribution and the robustness of the proposed GBLCA optimization method and its capability to attain optimum value or very close to it in almost all runs.

**Table 3:** Statistical Significance of GBLCA after 50 runs.

No. of Task	Best	Worst	Mean	Median	Mode	Standard Deviation
200	57	71	64.12	64	64	2.00155
400	91	105	97.66	98	98	2.02419
600	161	173	165.18	165	165	1.336519
800	239	251	242.28	242	242	0.597099
1000	313	324	317.72	318	317	1.25085
1200	445	462	454.98	456	455	0.78728
1400	573	594	581.40	580	581	0.568755
1600	677	691	686.32	685	684	1.22577
1800	789	812	796.48	797	794	1.00694
2000	871	893	888.96	891	887	0.77677

Table 4 presents the PIR (%) on makespan of the GBLCA as it relates to the MINMIN, MAXMIN, GA and the ACO schedulers. It shows that the proposed GBLCA produces 46.41%, 37.98%, 21.70% and 14.44% makespan improvements on the MINMIN, MAXMIN, GA and the ACO respectively. This implies that the proposed GBLCA performs better in terms of makespan minimization in the IaaS Cloud computing environment.

**Table 4:** Performance Improvement Rate (%) on Makespan.

	MINMIN	MAXMIN	ACO	GA	GBLCA
**Total Makespan**	6287	5925	5226	4914	4294
PIR% Over MINMIN		6.11	20.30	27.94	46.41
PIR% Over MAXMIN			13.37	20.57	37.98
PIR% Over ACO				6.35	21.70
PIR% Over GA					14.44

**Fig 6:**
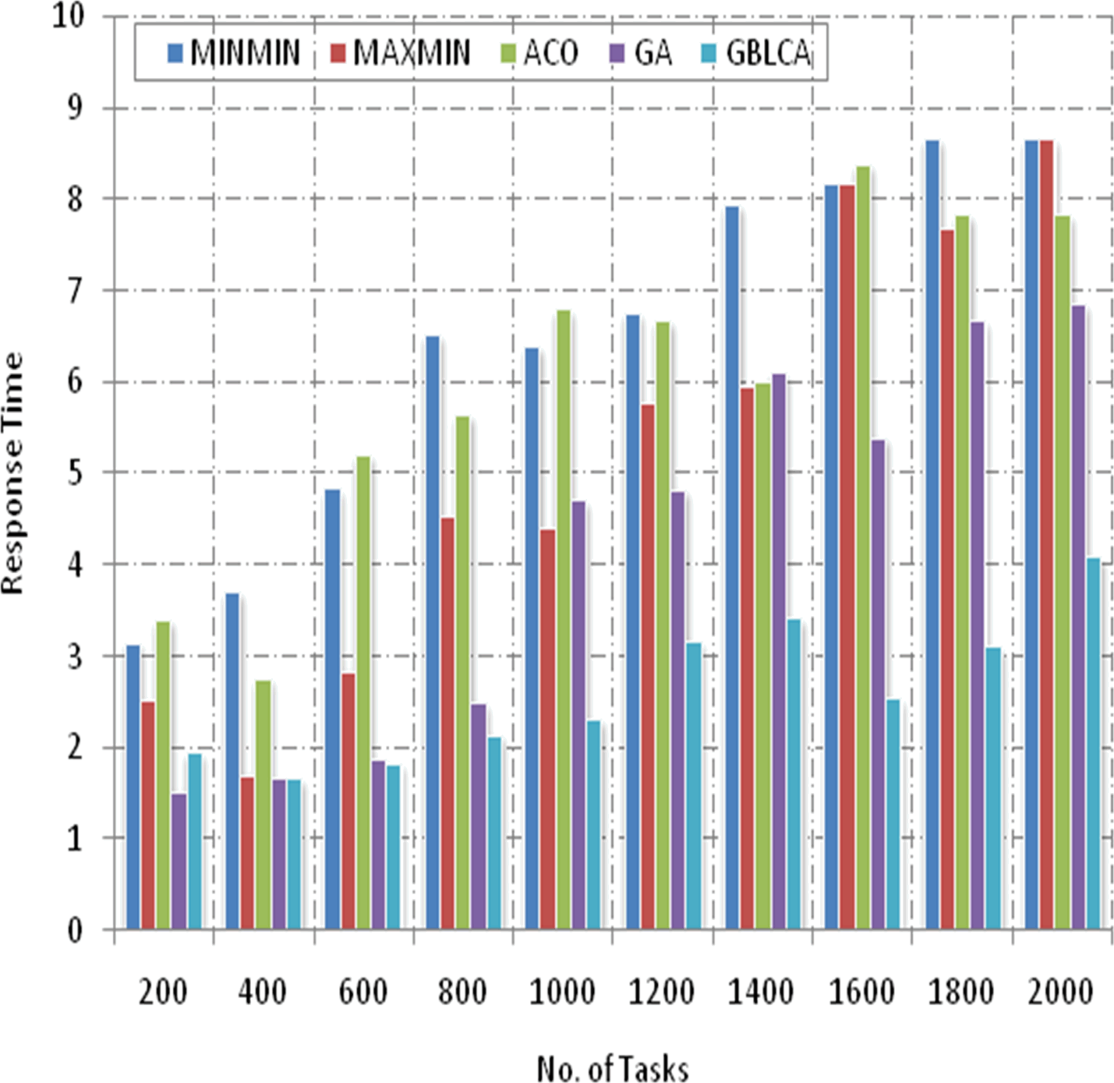
Response Time.

Fig 6 shows that the average response time for all the five algorithms i.e. MINMIN, MAXMIN, GA, ACO and GBLCA relatively increases with corresponding increase in the number of scientific application tasks submitted for execution in the IaaS cloud computing environment. The MINMIN, MAXMIN and ACO scheduling algorithms produce higher response times as compared to GBLCA throughout the scheduling process.

Conversely, the GA outperformed all other algorithms at the beginning of the experiment (that is at 200 and 400 cloudlets). As the number of cloudlets continues to increase above 400 cloudlets, the GBLCA begins to perform better than GA as well.

## Conclusions and Recommendations

This paper presents and discusses the experimental simulation results based on the proposed GBLCA. The experiments are designed and implemented using the CloudSim simulation framework as the Cloud Computing environment. Comparatively measured against the MINMIN, MAXMIN, GA and ACO, the novel proposed GBLCA shows greater level of performance in terms of makespan time and response time during secured global scientific application tasks scheduling in the IaaS Cloud Computing environment. In the proposed GBLCA scheduling technique, the tasks scheduling takes place at the highest level of cloud computing architecture and such it is Global. The purpose of this technique is to address the non-deterministic polynomial time problem of secure global task scheduling in the IaaS Cloud computing system. The GBLCA technique is designed to optimally map cloud users’ task applications to the scares on-demand cloud computing resources such as the cloud virtual infrastructures. However, the GBLCA tasks scheduling technique do not considers local job scheduling within the individual operating systems architecture in the form of operating systems processes.

The GBLCA has significantly attains a remarkable mapping of the LCA optimization algorithm and the Cloud computing scientific application tasks scheduling system. This is evaluated through experimental simulation results and comparison with other state-of-the-art tasks scheduling techniques which include heuristics (MINMIN and MAXMIN) and metaheuristics (GA and ACO). The results obtained shows that the proposed GBLCA generate 46.41%, 37.98%, 21.70% and 14.44% makespan performance improvement rate on the MINMIN, MAXMIN, GA and the ACO respectively. It also posted a significant reduction in the response time during tasks execution in this environment as measured in relation to the comparison scheduling techniques. This implies that the proposed GBLCA has performs better than the comparative schemes under consideration. However, local trappings at high level iterations is still a possibility as it cannot be totally eradicated and also only independent tasks are considered in the experiment.

A future research can also be done towards the implementation of these proposed techniques in the real cloud environment to investigate user’s satisfaction. The application of the GBLCA scheduling technique in the parallel computing system should be carried out as a further research. This research can also be extended to suggest that the GBLCA should be use to investigate resource allocation and provisioning issues in the cloud, grid and parallel computing environment. Also, hybridization of the algorithm with other metaheuristic optimization techniques should be explored. This is because of the promising performance of the algorithm in the distributed system environment.
